# Evaluation of health risk of glyphosate pesticide intake via surface and subsurface water consumption: A deterministic and probabilistic approach^✰^

**DOI:** 10.1016/j.mex.2023.102369

**Published:** 2023-09-07

**Authors:** Parichehr Pakzad, Ensiyeh Taheri, Mohammad Mehdi Amin, Ali Fatehizadeh

**Affiliations:** a Department of Environmental Health Engineering, School of Health, Isfahan University of Medical Sciences, Isfahan, Iran; b School of Health, Isfahan University of Medical Sciences, Isfahan, Iran; c Environment Research Center, Research Institute for Primordial Prevention of Non-Communicable Disease, Isfahan University of Medical Sciences, Isfahan, Iran

**Keywords:** Organophosphorus pesticide, Monte Carlo simulation, River and well, Isfahan, Human health risk assessment

## Abstract

As the usage of pesticides for both agricultural and non-agricultural uses increases, it is more important than ever to employ probabilistic methods rather than deterministic ones to calculate the danger to human health. The current work demonstrates the application of deterministic and probabilistic approaches to assess the human health risk related to glyphosate during the consumption of surface and groundwater by different population groups. To that aim, the concentration of glyphosate pesticide in the surface and groundwater was measured and human health risk for three population groups including children, teens, and adults was evaluated. Overall, the probabilistic approach via Monte Carlo simulation showed a valid result for the estimation of human health risk and determination of dominant input parameters.

• The health risk of glyphosate exposure during water consumption for various population groups were evaluated using deterministic and probabilistic methods.
• The modeling is performed by Crystal Ball (11.1.2.4) software, as open access software, and requires a limited number of inputs.
• The probabilistic method could reliably assess the risks of glyphosate by considering the variability and uncertainty in input variables.

## Graphical abstract

**Figure:**
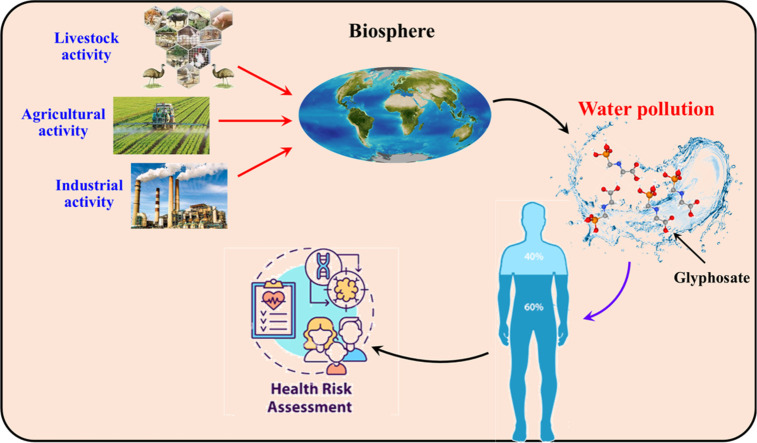

Specifications table

**Table:** 

Subject area:	Environmental Science
More specific subject area:	Risk characterization
Name of your method:	Human health risk assessment
Name and reference of original method:	[1], J.R. Fowle III, K.L. Dearfield, Risk Characterization: Handbook, EPA 100-B-00-002 Washington, DC 20460 (2000).
Resource availability:	Oracle Crystal Ball version 11.1.2.4 or later: https://www.oracle.com/applications/crystalball/

**Method details**

## Backgrounds

Due to the extensive use of pesticides for both agricultural and non-agricultural purposes and the fact that many of them have a half-life of several months, which allows them to remain for a long time in the environment, pesticide residues can be found in surface and subsurface water resources [2,3]. Pesticide chemicals' physicochemical characteristics such as their solubility in water and organic solvents and their octanol-water partition coefficients determine their amount of leaching [2]. The majority of pesticides that are released into the environment are thought to be harmful agents, and recently discovered toxicological interactions (mutagenicity, carcinogenicity, effects of environmental endocrine disruptor chemicals (EDCs) on hormones, immunomodulatory effects, etc.) have also been discovered [2,4]. Since it can be used to control a variety of plants, glyphosate (N-(phosphonomethyl)glycine) is one of the most widely used organophosphorus pesticides worldwide [5]. Its market is also expanding as more glyphosate-tolerant (GT) transgenic crops are being grown [6]. The groundwater ubiquity score (GUS) index for glyphosate, which shows the potential of pesticides to migrate into groundwater, is 1.5, indicating that the risk for high concentrations of this pesticide in the groundwater is low [7]. However, several studies already reported the presence of glyphosate in surface and groundwaters [8], [9], [10].

Models for assessing health risks can be used to provide systematic quantitative and semi-quantitative descriptions of the possible impacts of exposure to contaminants on human or environmental health [11]. Most conventional health risk assessments incorporate several assumptions to arrive at a conservative point estimate of risk [12,13]. This approach has several drawbacks, including (i) the fact that risk assessors and risk managers often combine moderate, conservative, and worst-case assumptions, making it impossible for them to grasp the degree of conservatism in an assessment. Point estimates may be difficult for risk managers and the general public to understand because current risk assessments frequently do not analyze uncertainty sufficiently, (ii) risk assessments may account for possibilities that will happen infrequently (if ever) by setting the bias high enough to smother the uncertainty for each of many variables, but not necessarily all of the variables, and (iii) because many of the input variables are at or close to their maximum, using conventional sensitivity analyses to calculate the uncertainties in the final point estimates is basically pointless [12]. On the other hand, due to its inherent natural variability, a probabilistic method can be an excellent predictive tool to achieve an indirect calculation of the health risk assessment [13].

## Case study area

Isfahan City is located in the center part of Iran and Its geographical coordinates are 32°38′41″N and 51°40′03″E, with a 1574 m elevation above sea level. The Zayanderud River provides the city of Isfahan's drinking water, which is then processed at the Baba Sheikh Ali water treatment plant before being distributed throughout the city. Additionally, the lack of water for public consumption, particularly during summer months, was made up for by wells in the city of Isfahan. In the spring of 2022, water samples from the Zayanderud River as surface water (5 points) and active dug wells as groundwater (6 points) were taken. We performed triplicate measurements for each sample and the data are present as a Mean±SD. To get accurate samples, the proposed standard criteria from the US-Geological Survey's Techniques of Water-Resources Investigations were taken into consideration [14]. In summary, sampling sites of river are located (i) at or near a stream gaging station, (ii) in straight reaches having uniform flow, (iii) far enough above and below confluences of streamflow or point sources of contamination, (iv) in reaches upstream from bridges or other structures, (v) in unidirectional flow without eddies, and (vi) at or near a transect in a reach where other data are collected. In the case of groundwater, wells must meet the following requirements: (i) without purging; (ii) continuous water production; (iii) without exposing the sampled water to atmospheric gasses; (iv) stabilized well criteria based on field measurements; and (v) the water drawn from the well must not pass through holding tanks or chemical treatments. The water quality of surface and groundwater as input data to assess the human health risk is summarized in Table 1.

**Table 1:** Characteristics of water samples.

Parameters	Value (Mean±SD)	
	Surface water	Groundwater
Glyphosate (µg/L)	1.32 ± 0.13	0.69 ± 0.17
pH	7.56 ± 0.15	6.97 ± 0.1
Dissolve oxygen (mg/L)	6.22 ± 0.4	2.97 ± 0.4
Electrical conductivity (µS/cm)	136.92 ± 4.1	459.0 ± 51.8
Nitrate (mg/L as N)	1.0 ± 0.25	0.58 ± 0.13
Total hardness (mg/L)	151.4 ± 2.97	229.67 ± 12.7
Total alkalinity (mg/L)	147.2 ± 6.42	100.33 ± 4.1

## Instrumental analysis

**Fig. 1:**
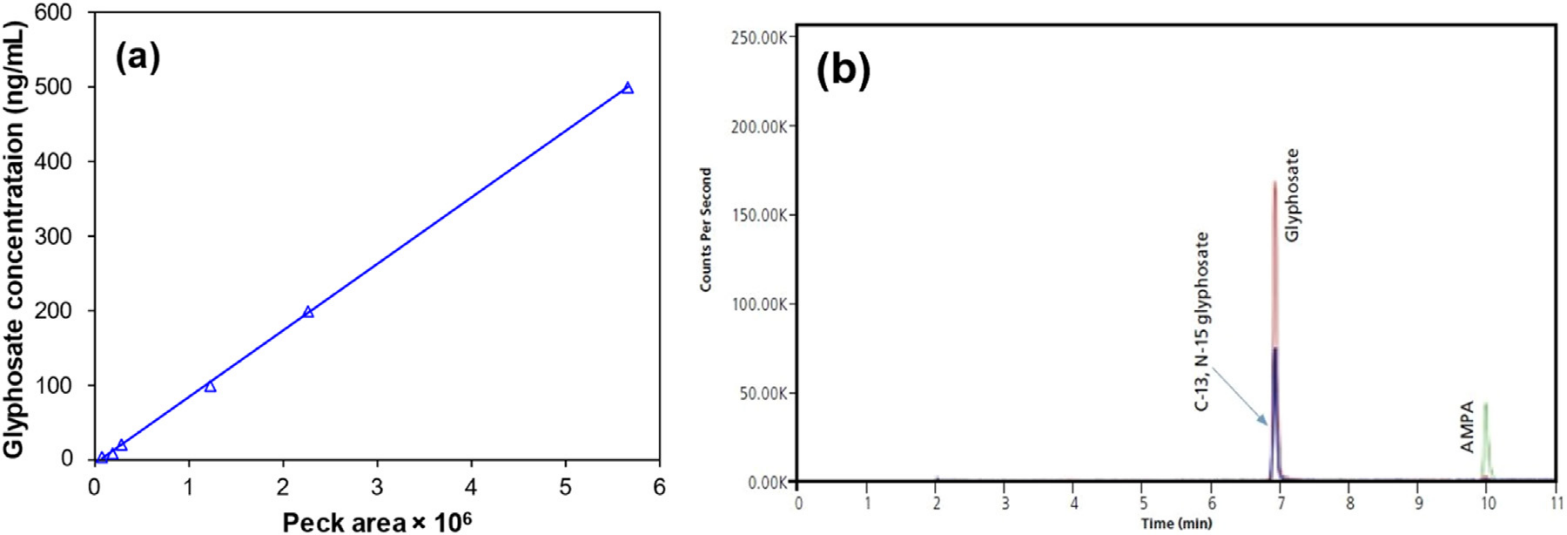
Calibration curve of glyphosate (5–500 ng/mL, *n* = 3, and R^2^ = 0.998) and (b) LC/MS elution profile of glyphosate (red trace).

To minimize potential glyphosate interactions with matrix components, 1 mL of each collected water sample was acidified with 10 µL of 1 M formic acid. Then, 50 µL of borate buffer (5% tetraborate solution) and 50 µL of fluorenylmethyloxycarbonyl chloride in acetonitrile (FMOC) (10 mg/mL) were added to 100 L of samples to derivatize, and then allow the reaction to take place overnight at room temperature. Thereafter, the reaction was then stopped by adding phosphoric acid (500 µL of a 2% solution) to the solution and removing the excess FMOC with methanol (300 µL) [15]. The concentration of glyphosate pesticide in the samples was determined with HPLC equipped with a quaternary pump, degasser and autosampler (Agilent 1200 series, Santa Clara, CA, USA) and coupled to a quadrupole mass spectrometer (Agilent 6130 series, Wilmington, USA) with an Ultraspray ESI. In typical measurement, 4 µL of the samples were injected in the partial fill mode, and the flow rate was set at 0.4 mL/min. The separation was performed in a 100 mm BEH C18 XP column (130 Å, 2.5 mm particles, 2.1 mm inside diameter, Waters, Milford, MA, USA) interfaced to a 5 mm XBridge BEH C18 VanGuard Pre-column (130 Å, 2.5 mm particles, 2.1 inside diameter, Waters, Milford, MA, USA) with 5 mM ammonium formate in water (A) and acetonitrile (B) as mobile phase in the gradient mode. The gradient program was as follows: 2 min (A: 80% and B: 20%), 7 min (A: 60% and B: 40%), and 6 min (A: 10% and B: 90%). The settings for MS detector include a capillary exit voltage of −70 V, drying gas flow of 8 mL/min at 300 °C, nebulizer pressure of 35 psi, sheath gas flow of 3 mL/min at 150 °C, dwell time of 5.79 ms, and negative mode with selected ion monitoring (SIM) mode of 390 amu [16,17]. The limit of detection (LOD) and the limit of quantification (LOQ) of glyphosate were 0.01 and 0.03 ng/mL, respectively (Fig. 1). Nitrate concentration in water samples was determined using a colorimetric approach (DR 5000^TM^ UV–Vis Spectrophotometer, Hach, Germany), as well as total hardness and total alkalinity were measured by a titration method. Other parameters of water samples including solution pH, dissolve oxygen, and electrical conductivity were quantified by precalibrated multi-parameter meter devise (HQ40d, Hach, Germany). All test methods were adopted from the “Standard methods for the examination of water and wastewaters” [18].

## Calculations

Calculating the level of human exposure to glyphosate through the oral route of pollutants exposure to the human body is important to evaluate the human health risk. Eq. (1) was used to predict the daily intake of glyphosate (EDI) for deterministic simulation of health risk [19], where C_i_ is average concentration of glyphosate in water (mg/L), IR is ingestion rate of water (L/d), EF and ED are exposure frequency (d/year) and exposure duration (year), respectively, BW is average body weight (kg), and AT is the averaged exposure time (day). For each exposed group, the typical value of mentioned parameters is shown in Table 2.

(1) $$EDI=\frac{C_{i}\times IR\times EF\times ED}{BW\times AT}$$

**Table 2:** Typical values for estimation of human health risk.

Parameter	Population group			Reference
	Children	Teens	Adults	
IR (L/d)	0.78	2	2.5	[1]
EF (day/year)	350	350	350	[20]
ED (year)	4	13	40	[1]
BW (kg)	15	50	80	[21]
AT (day)	1400	4550	14,000	[22]
Reference dose (RfD) (mg/kg-day)	0.1	0.1	0.1	[23]

The ratio of exposure to glyphosate is known as the hazard quotient (HQ), which is calculated using Eq. (2) to determine the daily intake of glyphosate (via drinking water consumption). If the HQ is less than 1, the detrimental consequences of exposure cannot be anticipated, and if it is greater than 1, there may be a concern for human health [11].

(2) $$HQ=\frac{EDI}{RfD}$$

The lack of knowledge of the population characteristics and environmental conditions makes data uncertainty a critical component of the evaluation of the risks to human health. The chance of uncertainty in risk assessment can grow with the implementation of a deterministic technique with a single-point value for each variable. Instead, reducing the likelihood of uncertainty by applying a probabilistic approach to the set of data. The Monte Carlo simulation is one such technological technique. After developing a deterministic model, the simulation replaces point estimates with assumptions about probability distributions and predicts the distribution of the output. To determine how risky the scenario is, the expected output distribution is employed. The parameter distributions for the population groups in the Monte Carlo simulation are listed in Table 3. To test the convergence and stability of the results for the highly skewed distributions, the Monte Carlo simulation software was run 100,000 times, and the results were compared to independent simulations. Additionally, a Monte Carlo simulation was used to conduct a sensitivity analysis to evaluate the relative significance of each input parameter.

**Table 3:** Parameter distributions in Monte Carlo simulation.

Parameter	Population group			Probability distribution	Reference
	Children	Teens	Adults		
IR (L/d)	0.51 ± 0.14	1.12 ± 0.27	1.23 ± 0.27	Log normal	[24]
EF (day/year)	365	365	365	Fixed value	[25]
ED (year)	1–7	8–25	26–80	Uniform	[11]
BW (kg)	16.41 ± 3.78	46.25 ± 10.16	77.45 ± 13.60	Log normal	[19,25]
AT (day)	1400	4550	14,000	Fixed value	[22]
RfD (mg/kg-day)	0.1	0.1	0.1	Fixed value	[23]
Glyphosate in surface water (µg/L)	1.32 ± 0.13	1.32 ± 0.13	1.32 ± 0.13	Normal	Present study
Glyphosate in groundwater (µg/L)	0.69 ± 0.17	0.69 ± 0.17	0.69 ± 0.17	Normal	Present study

### Software

Implementation of human health risk assessment by Monte Carlo simulation modeling was done using the Crystal Ball spreadsheet (11.1.2.4) in Microsoft Excel software.

### Method validation

**Fig. 2:**
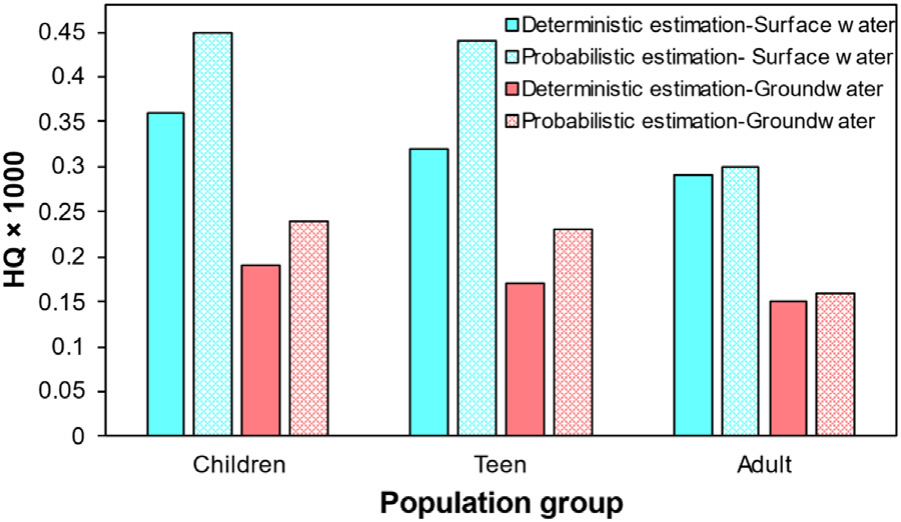
Comparison of estimated HQ by deterministic and probabilistic approach.

For children, teens, and adults, the HQ values were calculated using a deterministic method using Eqs. (1) and (2) to assess the possible human health risk of glyphosate during surface and groundwater consumption. As can be seen in Fig. 2, the HQ values for all population groups are less than 1, indicating that there is no risk to human health from glyphosate during surface and groundwater consumption. However, the findings showed that children have higher HQ levels than teens and adults.

**Fig. 3:**
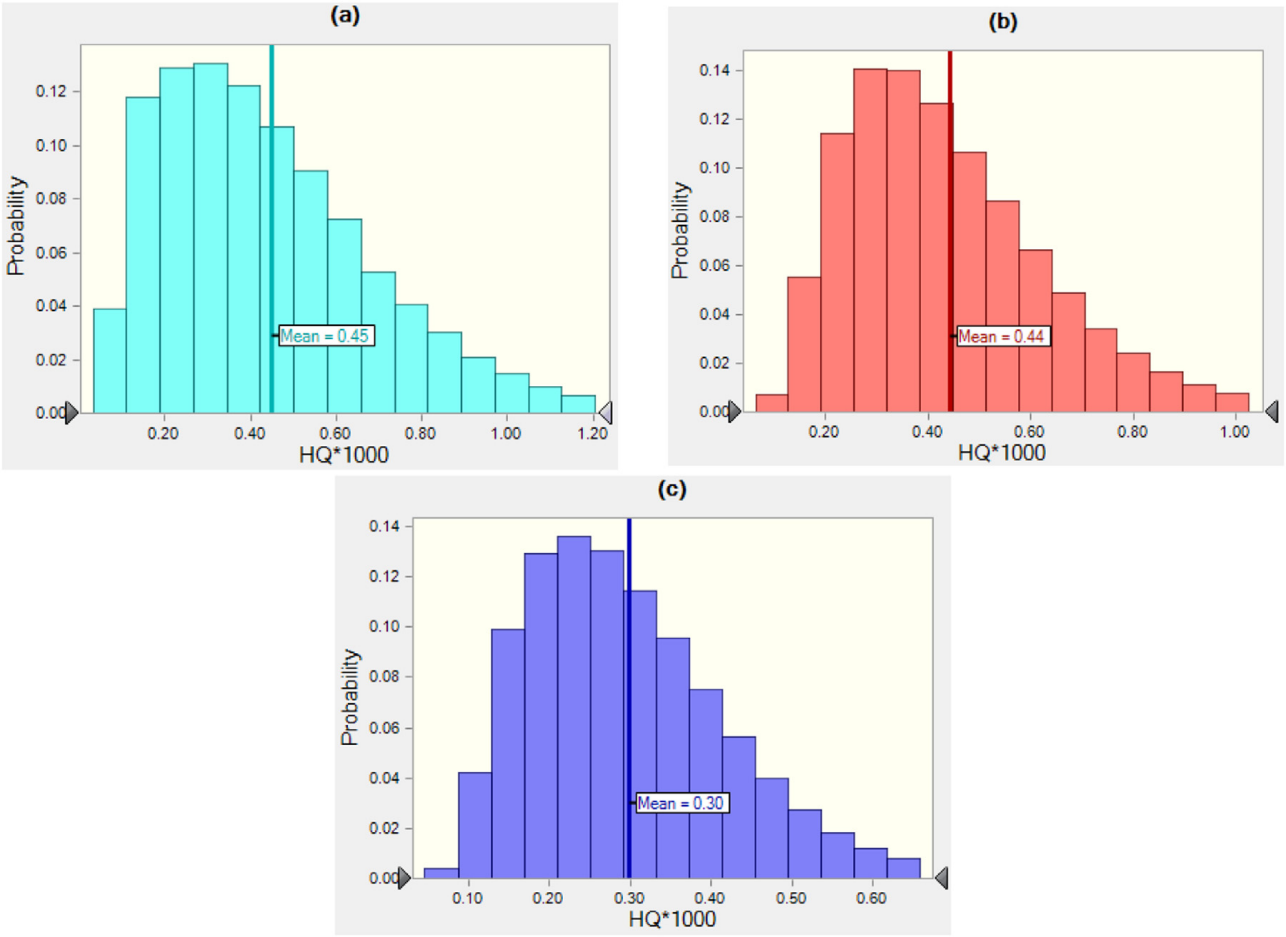
Histograms of the uncertainty analysis of HQ related to glyphosate during consumption of surface water, (a) children, (b) teen, and (c) adult population.

**Fig. 4:**
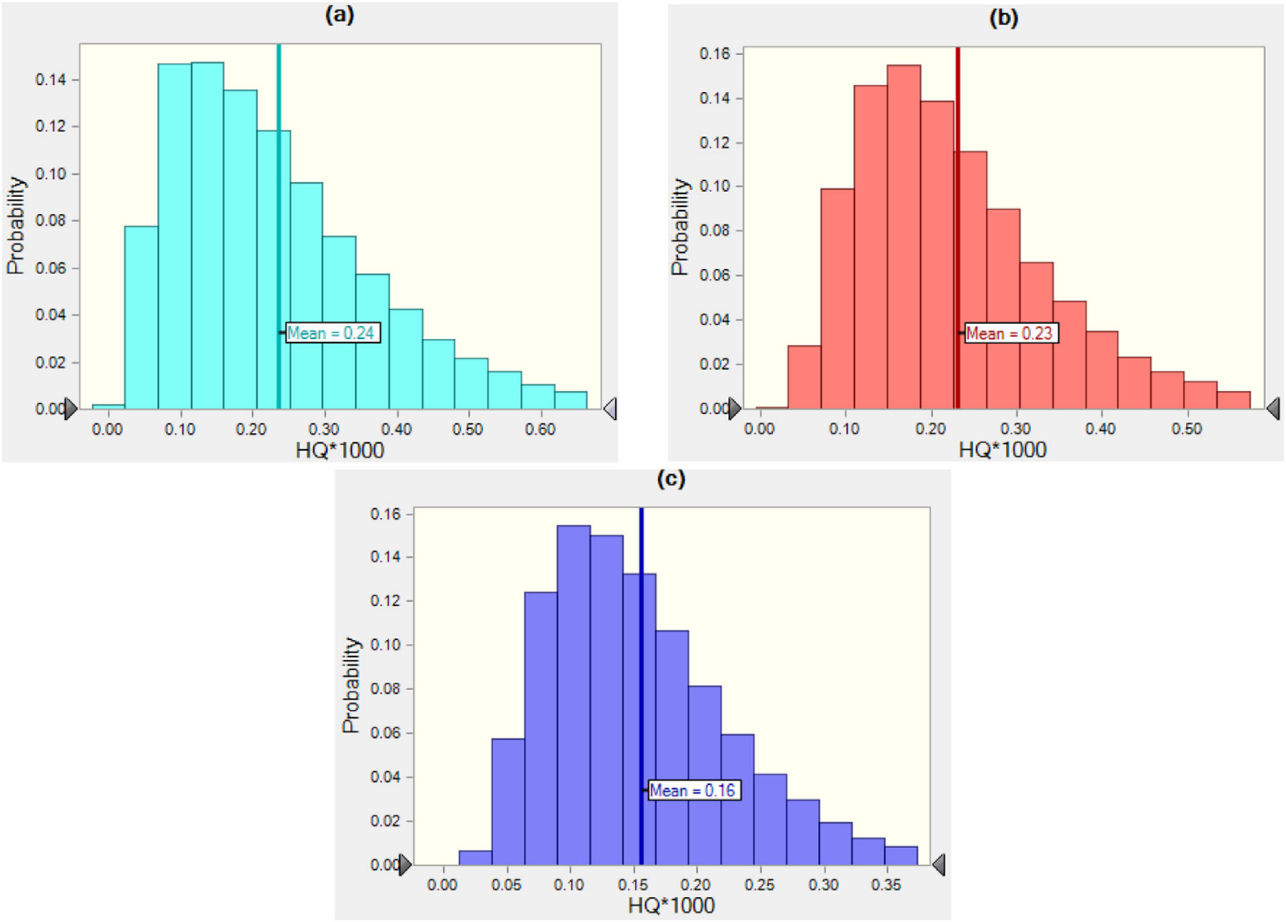
Histograms of the uncertainty analysis of HQ related to glyphosate during consumption of groundwater, (a) children, (b) teen, and (c) adult population.

Also, we performed the estimation of HQ by probabilistic approach with 100,000 repeats using the Crystal Ball (11.1.2.4) software to validate the results of the Monte Carlo simulation. The histograms for simulating HQ results for various population groups and water sources are shown in Figs. 3 and 4. From the histogram, the mean values of HQ were extracted and compared to the estimated HQ by deterministic method (Fig. 2). As displayed, the estimated HQ value using the deterministic and probabilistic approaches are relatively closed, indicating that the Monte Carlo simulation may be utilized successfully for HQ estimation.

**Fig. 5:**
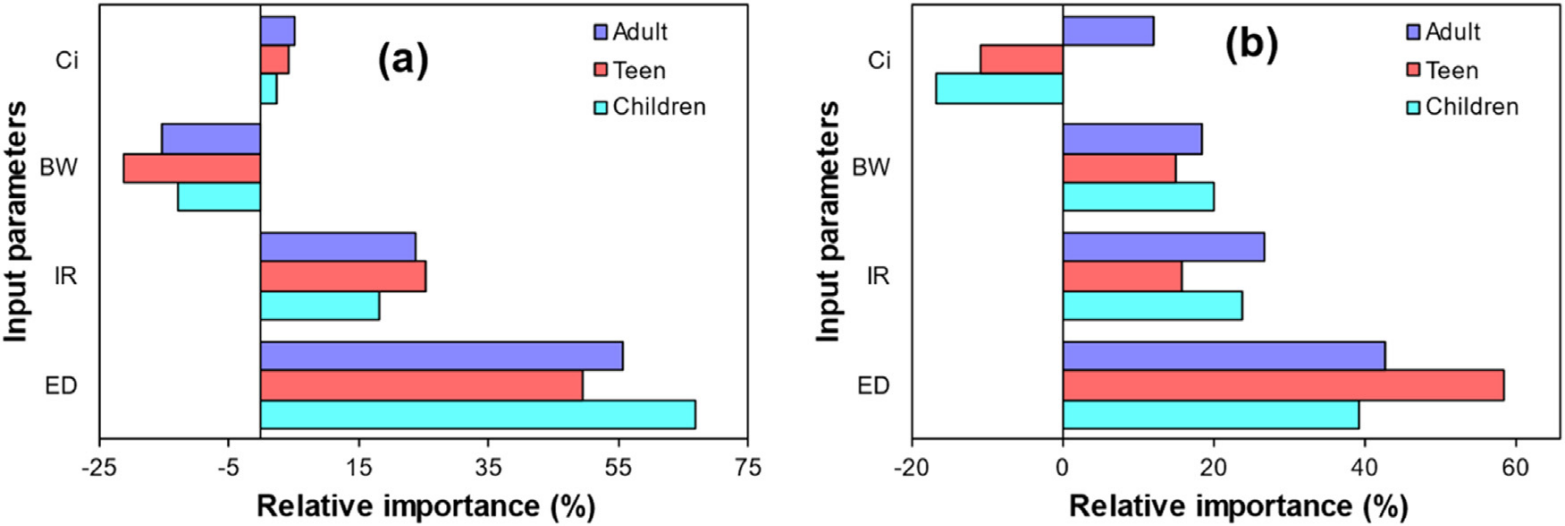
Sensitivity analysis of different age groups exposed to glyphosate, (a) surface water, and (b) groundwater.

As previously mentioned, due to a lack of knowledge regarding the true value of input parameters, uncertainty is one of the main problems associated with health risk estimation using deterministic methods. Since some of the parameters are more important than others during the human health risk assessment, the identification of the dominant parameter by sensitivity analysis can be helpful for better understanding and consequently, proper management of drinking water resources. The sensitivity analysis shows how the changing of the input parameters affects the uncertainty of the final result. Fig. 5 shows the sensitivity analysis of the input parameters used for HQ estimation. As displayed, the exposure duration is the dominant factor influencing health risk for different groups of the population.

### Comparison of the deterministic and probabilistic approaches

In general, point values are entered into a given deterministic approach, neglecting the variability in the input variables, and resulting in biases in the ensuing human health risk assessment results [26]. Experts from business, government, and academia have created, assessed, and improved probabilistic models to better identify and comprehend the variety of potential cumulative and aggregate exposures and health concerns. The stochastic selection of model input values based on actual data distributions, population-based assessments, and calendar-based (365 days) exposure calculations are key elements of probabilistic techniques [27]. Numerous models, including the Stochastic Human Exposure and Dose Simulation Model (SHEDS), the Cumulative and Aggregate Risk Evaluation System (CARESs), Calendex™, and ConsExpo, are currently being assessed to predict pesticide exposures using sparse data inputs [28,29]. The Monte Carlo simulation method considers inherent randomness in input variables, adopting appropriate probability distributions for meaningful results. It defines assumptions, fits data with probability distributions, forecasts using risk assessment models, and analyzes risk at each iteration. Sensitivity analysis estimates exposure variable contribution to total health risk, identifying critical impact factors [30].

The deterministic method was used to calculate the HQ point values in the assessment of HQ related to glyphosate in surface water and groundwater for population groups, and the probabilistic approach was used to derive the HQ probability distributions by considering the variability and uncertainty in the input variables. Both approaches gave detailed information on the HQ level and significant exposure paths. However, because the percentiles in the probability distribution of HQ produced with the probabilistic technique were irregular, the HQ values estimated with the deterministic approach might understate the risks of glyphosate to human health (Fig. 2). This work confirms the HQ estimation by probabilistic approach via Monte Carlo simulation is valid for the estimation of human health risk and determination of dominants input parameters. Using deterministic and probabilistic approaches, Zhang et al. [30] evaluated the human risks of 7 polycyclic aromatic hydrocarbons (PAHs) for groups of different ages and sexes and found that the deterministic approach could overestimate or underestimate the true risks of PAH, whereas the probabilistic method could reliably assess the risks of PAH.

## Ethics statements

The procedure was subject to approval from the ethics committee of the Isfahan University of Medical Sciences (Ethics code: IR.MUI.RESEARCH.REC.1398.26).

## CRediT authorship contribution statement

**Parichehr Pakzad:** Investigation. **Ensiyeh Taheri:** Software, Validation, Data curation, Writing – original draft. **Mohammad Mehdi Amin:** Data curation, Visualization, Writing – review & editing. **Ali Fatehizadeh:** Conceptualization, Supervision, Methodology, Writing – review & editing.

## Declaration of Competing Interest

The authors declare that they have no known competing financial interests or personal relationships that could have appeared to influence the work reported in this paper.

## Data Availability

Data will be made available on request.
